# Evaluation of Taterapox Virus in Small Animals

**DOI:** 10.3390/v9080203

**Published:** 2017-08-01

**Authors:** Scott Parker, Ryan Crump, Hollyce Hartzler, R. Mark Buller

**Affiliations:** Department of Molecular Microbiology and Immunology, Saint Louis University School of Medicine, 1100 South Grand Boulevard, St. Louis, MO 63104, USA; rcrump1313@gmail.com (R.C.); hollyce@gmail.com (H.H.); mark.buller@gmail.com (R.M.B.)

**Keywords:** smallpox, variola, taterapox, monkeypox, orthopoxvirus, SCID, footpad, intranasal

## Abstract

Taterapox virus (TATV), which was isolated from an African gerbil (*Tatera kempi*) in 1975, is the most closely related virus to variola; however, only the original report has examined its virology. We have evaluated the tropism of TATV in vivo in small animals. We found that TATV does not infect *Graphiurus kelleni*, a species of African dormouse, but does induce seroconversion in the Mongolian gerbil (*Meriones unguiculatus*) and in mice; however, in wild-type mice and gerbils, the virus produces an unapparent infection. Following intranasal and footpad inoculations with 1 × 10^6^ plaque forming units (PFU) of TATV, immunocompromised *stat1^−/−^* mice showed signs of disease but did not die; however, SCID mice were susceptible to intranasal and footpad infections with 100% mortality observed by Day 35 and Day 54, respectively. We show that death is unlikely to be a result of the virus mutating to have increased virulence and that SCID mice are capable of transmitting TATV to C57BL/6 and C57BL/6 *stat1^−/−^* animals; however, transmission did not occur from TATV inoculated wild-type or *stat1^−/−^* mice. Comparisons with ectromelia (the etiological agent of mousepox) suggest that TATV behaves differently both at the site of inoculation and in the immune response that it triggers.

## 1. Introduction

Because smallpox no longer circulates in human populations, the licensure of new therapeutics and prophylactics is through efficacy testing in animal models as outlined in the Animal Rule (United States Code of Federal Regulations title 21, part 314, subpart I, Federal Register 2002). Although vaccinia (VACV), cowpox (CPXV) and ectromelia (ECTV) viruses in mice [1,2,3,4]; VACV/rabbitpox (RPXV) in rabbits [5,6]; and monkeypox (MPXV) virus in cynomolgus and rhesus monkeys [7,8,9,10,11,12], 13-lined ground squirrels [13,14], prairie dogs [15,16,17], dormice [18], and mice [19,20,21,22,23,24] have been used to model smallpox, no one model faithfully recapitulates human disease [25]. Taterapox virus (TATV) is an orthopoxvirus and is generally considered to be the closest known phylogenic relative of variola virus (VARV, also an orthopoxvirus) based on: restriction mapping; viral linear genome sequencing; comparisons of conserved genes in poxviruses, chordopoxviruses and orthopoxviruses; and comparison of the F10L/F12L open reading frame (ORF) from VACV-Copenhagen. Here, we investigated TATV as a surrogate for VARV in vivo [26,27].

Despite the similarity between VARV and TATV, only one scientific report, written in 1975, has been published that directly examines the virology of TATV [28]. TATV was isolated from an apparently healthy and wild gerbil (*Tatera kempi* or *Gerbilliscus kempi*) caught in northern Dahomey (now Republic of Benin) in 1968. The virus was noted to grow well on chorioallantoic membranes (CAMs) and produce pocks of similar sizes to those of VARV and had a ceiling temperature of 38 °C—also similar to VARV. Moreover, TATV produced a cytopathic effect similar to that of VARV, but distinct from ECTV, MPXV, RPXV, CPXV, or VACV in Vero, LLC-MK2, GMK-AH, and RK-13 cell lines. Intracranial (IC) or intraperitoneal (IP) inoculation of the Mongolian gerbil (*Meriones unguiculatus*) with TATV suspension revealed no obvious signs of disease. No virus could be recovered from the spleens, livers, or kidneys of gerbils sacrificed on Days 14 and 19 post infection (p.i.). It was noted that one rabbit inoculated intradermally (ID) developed a local lesion which ulcerated and crusted over. In three other rabbits, reddened indurated areas occurred at the site of inoculation but resolved without ulceration or crusting. No hemorrhagic or secondary lesions were observed on any of the rabbits. One monkey (*Macaca mulatta*) was inoculated via the intramuscular (IM) and intranasal (IN) routes and developed a fever of 104 °F (40 °C); however, no lesions were observed and no viremia could be detected. The monkey did have increased levels of hemagglutinin inhibition antibodies to orthopoxviruses (OPVs) and the monkey resisted a MPXV inoculation at 10 weeks p.i. (route unspecified). No virus was isolated when liver, spleen, kidney or pancreas suspensions from the inoculated animals were inoculated onto CAMs or Vero cells [28]. Thus, similar to VARV, no animal model tested to date supports robust TATV replication [29].

In this report, we extend our understanding of the tropism of TATV in small animal models. In particular, we have examined the infection and pathology of TATV in the Mongolian gerbil, a species of African dormouse (*Graphiurus kelleni*) and several wild-type and immunocompromised mouse strains. We found that TATV induces seroconversion in the gerbil and mice; however, in wild-type mice and gerbils the infection is unapparent. Immunocompromised *stat1^−/−^* mice did show signs of disease but experienced no mortality; however, SCID mice were susceptible to both IN and footpad (FP) infections. Furthermore, SCID mice were capable of transmitting TATV to C57BL/6 and C57BL/6 *stat1^−/−^* animals. Following a FP inoculation, TATV replicated but induced a different cytokine response to that of ECTV. This response is likely to have contributed to the failure of TATV to move to the draining lymph node.

A subsequent paper will present data pertaining to in vitro studies of TATV which reveal further insights into the biology of this virus.

## 2. Materials and Methods

### 2.1. Animals

The Institutional Animal Care and Use Committee at Saint Louis University School of Medicine approved all experimental protocols (protocol 2082). A/Ncr and C57BL/6 mice were purchased from the National Cancer Institute (Frederick, MD, USA). Mongolian gerbils and 129, SCID (BALB/c background), SCID (SKH1 background) and SKH1 mice were acquired from Charles River Laboratories (Wilmington, MA, USA). Dormice were acquired from an in-house colony [18]. The 129 *stat1^−/−^* mouse strain was acquired from Taconic (Hudson, NY, USA), it was originally developed in the laboratory of Robert Schreiber at Washington University School of Medicine (St. Louis, MO, USA) [30]. C57BL/6 mice carrying a *stat1^−/−^* mutation were provided by Michael Holtzman (Washington University School of Medicine) who acquired them from Joan Durbin (New York University School of Medicine, NY, USA) [31].

All experimental and animal procedures were completed at animal biosafety level-3 (ABSL-3) where animals were housed in filter-top microisolator cages. A standard rodent diet (Teklad Global 18% Protein Rodent Diet, Envigo, Huntingdon, UK) and water were provided ad libitum. Corn cob bedding was provided in each cage where no more than 5 animals were housed. All animals were acclimatized for at least one week prior to infection. Animals were 6–12 weeks in age and experiments consisted of 4–5 animals per group (unless otherwise stated). Experiments were performed at least twice.

For IN infection, mice and dormice were anesthetized by IP injection of 9 mg/mL ketamine HCl (90 mg/kg) and 1 mg/mL xylazine (10 mg/kg) at a ratio of 0.1 mL/10 g body weight. Gerbils were anesthetized as above but with 6 mg/mL ketamine HCl (60 mg/kg) and 0.5 mg/mL xylazine (5 mg/kg). IN inoculations with 5 μL/nare of virus were used to seed the upper respiratory tract as described previously [32]. For FP infections, 10 µL/pad of virus was used, animals were briefly exposed to CO_2_:O_2_ (4:1) followed by injection with a 29 × 1/2 gauge needle.

### 2.2. Cells and Viruses

BS-C-1 cells (ATCC CCL 26) were grown in Dulbeccos’ modified eagle’s media DMEM (Lonza, Basel, Switzerland) containing 10% fetal calf serum (FCS) (Hyclone III, Logan, UT, USA), 2 mM l-glutamine (GIBCO, Grand Island, NY, USA), 100 U/mL penicillin (GIBCO), and 100 μg/mL streptomycin (GIBCO). Virus was purified through a sucrose cushion as described elsewhere [33]. Animals were sacrificed and bled by heart-sticks to determine viral titers. Tissues were removed and ground in phosphate buffered saline (PBS) (10% FBS *w*/*v*). Footpads were removed with a surgical blade (Henry Schein, Melville, NY, USA) and processed in the same manner as tissue. Following grinding, samples were freeze–thawed thrice interrupted by 20 s sonications. Virus infectivity was calculated by titration on BS-C-1 monolayers for seven days (TATV) or four days (ECTV) at 37 °C [34]. Plaques were visualized by addition of 0.5 mL 0.3% crystal violet/10% formalin to each well. Arithmetic means above the limit of detection (1 × 10^2^ PFU/mL) were calculated as plaque forming units (PFU)/g or PFU/mL [35].

TATV was a gift from Geoffrey Smith (Imperial College, London, UK). The virus was isolated from a wild gerbil (*Tatera Kempi*) caught in Dahomey (now Republic of Benin), Africa in 1968 [28]. Plaque purified isolates of TATV, ECTV-MOS and VACV-COP were propagated in BS-C-1 cells.

### 2.3. Psoralen Inactivation

TATV was mixed in a 24-well plate with 4′-aminomethyltrioxsalen (psoralen) (Sigma, St. Louis, MO, USA) to a final concentration of 10 µg/mL in a total volume of 200 µL. After a 10 min incubation at room-temperature (RT) the cover of the 24-well plate was removed and the plate was exposed to LWUV (high-intensity ultraviolet lamp 365 nm, 120 volts, 60 Hz, 1.05 amp; Spectronics Corp., Wesbury, NY, USA) at a distance of ~7 inches for 30 min with gentle agitation every 5 min. Aliquots were prepared and stored at −80 °C. After freezing, aliquots (and controls) were thawed and titered, as above. Psoralen-treated stocks yielded no plaques.

### 2.4. ELISAs

For mice, direct anti-OPV ELISAs were performed using lysates from BS-C-1 cells infected with VACV-WR. Clarified cell lysate was diluted in 50 mM carbonate-bicarbonate buffer (pH 9.6) at a 1:2500 dilution, and used to coat 96-well microtiter ELISA plates at 4 °C overnight. Plates were blocked with blocking buffer (PBS + 0.05% Tween 20 + 2% normal goat serum; Vector, Burlingame, CA, USA) at room temperature for 30 min, and serial dilutions of mouse sera were added to wells. Following incubation at room temperature for 1 h, wells were washed with PBS-T + 0.05% Tween 20. Bound antibody was detected by using biotin-conjugated goat anti-mouse IgG (Invitrogen, Carlsbad, CA, USA) at 1:2500 dilution followed by streptavidin-HRP (Invitrogen) at 1:4000 and orthophenylenediamine (0.4 mg/mL) in 50 mM citrate buffer (pH 5.0) as a chromogen. Optical density was measured at 490 nm. For dormice and gerbil serum a modified ELISA was used with a 1:30,000 dilution of HRP conjugated protein A/G (Thermo Scientific, Worcester, MA, USA).

### 2.5. In-Life Animal Assays, Cytokine Analysis and qPCR

Animals were weighed at least daily from the day of infection to Day 21 and then weekly until sacrifice. Mortality was scored daily. Moribund animals were sacrificed and their survival was scored one day later than sacrifice. For transmission studies, animals were bled for approximately 100 µL of blood by sub-mandibular (mice) or retro-orbital (gerbils and dormice) bleeds at two days before infection and confirmed to be ELISA negative for OPV antibodies. Index animals were inoculated and held for two days before being moved to cages containing contact animals. Animals were subsequently bled approximately every four weeks and checked by ELISAs for seroconversion. For cytokine assays, a bioplex cytokine assay kit (Bio-Rad, Hercules, CA, USA) was used to analyze 32 cytokines: interleukin (IL)-1a, IL-1b, IL-2, -3, -4, -5, -6, -7, -9, -10, -12p40, -12p70, -13, -15, and -17; LIF; vascular endothelial growth factor (VEGF); MIG; LIX; MIP-2; IP-10; M-CSF; GCSF; GM-CSF; interferon (IFN)-γ; eotaxin; KC; MCP-1; MIP-1a; MIP-1b; Rantes; and tumor necrosis factor (TNF)-α. Assays were run on a Luminex-100 (Luminex, Austin, TX, USA) and analyzed with xPONENT (Luminex) software. For qPCR, lymph nodes were removed to PBS 0.05% Triton X-100 and ground up to make a homogenate. The suspension was incubated at 4 °C overnight and then spun-down to remove debris. The 1.25 µL of homogenate/25 µL reaction was directly run using Omniklentaq buffer and enzyme according to the manufacturer’s instructions [36] (DNA Polymerase Technologies, St. Louis, MO, USA).

### 2.6. Microscopy

Light microscopy images were taken with a Zeiss (Oberkochen, Germany) dissecting microscope with a 3.2× objective lens. The images were captured using an Olympus (Tokyo, Japan) 5.1 megapixel C-5060 wide zoom camera and were processed in Microsoft Power Point (version 15; Redmond, WA, USA).

### 2.7. Histopathology

Tissues from IN and FP mice were collected for microscopic evaluation from moribund mice along with respective mock controls. The following tissues were collected in 10% neutral buffered formalin, fixed for 24 h and then transferred to 70% ethanol prior to trimming, processing and embedding in paraffin: adrenal gland, aorta, bone and bone marrow (femur and sternum), brain, cecum, colon, duodenum, esophagus, eye, gallbladder, harderian gland, heart, ileum, jejunum, kidney, larynx, liver, lung, lymph nodes (submandibular, mesenteric), mammary gland, nasal cavity, optic nerve, ovary, oviduct, pancreas, parathyroid, pituitary gland, rectum, salivary gland, skeletal muscle, skin, spinal cord, spleen, stomach, thymus, thyroid, tongue, trachea, urinary bladder, uterus with cervix, and vagina. Paraffin sections were stained with hematoxylin and eosin.

### 2.8. Statistics

*T*-tests were used to compare means between groups of animals and to determine the mean time to death. *T*-tests were also used to compare changes in viral titers and cytokine levels based on sample sizes of six tissue titers or cytokine preparations from different mice. Mortality rates were compared using the Fisher’s exact test. Blinded images were measured qualitatively using a scoring system. Throughout the manuscript, “significant” indicates *p* values < 0.05.

## 3. Results

### 3.1. TATV Infection of Immunocompetent Animal

TATV was originally isolated from a wild African gerbil, *Tatera kempi*, which are not available commercially or easily obtainable from Africa [28]. For this reason, we investigated the pathogenesis of TATV in the commercially available Mongolian gerbil, *Meriones unguiculatus*, which were inoculated by the FP or IN routes with 1 × 10^6^ PFU of TATV. Over a 60-day observation period, the inoculated gerbils lost no weight, showed no signs of morbidity and did not die, although inoculated animals seroconverted by Day 70 p.i. (Table 1). We next evaluated a second African rodent species, *Graphiurus kelleni* (dormouse), that has previously been used to study MPXV infections for susceptibility to severe disease [18]. No morbidity or mortality was observed following FP and IN inoculations with 1 × 10^6^ PFU of TATV and inoculated dormice failed to seroconvert up to 120 days p.i. We also investigated the lethality of TATV for different immunocompetent mouse strains. Infections of A/Ncr, SKH1, C57BL/6, CAST/EiJ, and 129 mouse strains with 1 × 10^6^ PFU of TATV via the FP and IN routes resulted in no weight-loss, morbidity or mortality, although all inoculated mice seroconverted by 60 days p.i. (Table 1). Furthermore, virus was not recovered from the livers, kidneys, spleens, and lungs of inoculated 129 mice sacrificed on Days 12 and 20 p.i. (data not shown). TATV IN inoculated hairless SKH-1 mice failed to present with rash under conditions where ECTV inoculated mice did (data not shown). Consistent with the lack of systemic disease, TATV was not detected in the livers, kidneys, spleens, and lungs of SKH1 mice sacrificed at Day 25 p.i. These studies extend the list of animal species that do not support robust TATV replication.

### 3.2. Inactivated TATV Induces Seroconversion

Except dormice, all of the immunocompetent animals seroconverted despite showing no overt signs of morbidity; furthermore, tissue titers from 129 and SKH1 mice were negative for TATV. Conventionally, seroconversion occurs following an active infection with associated viral replication; however, seroconversion can also occur when the immune system is exposed to viral antigen—reminiscent of the seroconversion observed following some vaccination protocols. To determine the cause of seroconversion we inoculated BALB/c mice via the IN or FP route with TATV (1 × 10^3^ PFU) and TATV that had been made replication deficient via psoralen inactivation (TATV-psoralen). At *T* = 28 days mice were bled and assayed for seroconversion by ELISA. At *T* = 35 days, mice were inoculated with a lethal dose of ECTV (1 × 10^3^ PFU) via the corresponding inoculation route and monitored for survival. As expected, mock-inoculated mice failed to seroconvert and experienced 100% mortality by eight days post ECTV inoculation (Table 2). Mice that were inoculated via the IN or FP route with TATV experienced seroconversion and were protected against the subsequent ECTV inoculation. Only one (1 of 3) mouse inoculated via the FP route with TATV-psoralen experienced seroconversion and this animal was protected against the subsequent ECTV inoculation; however, the animals that did not seroconvert experienced mortality by Day 7 post ECTV inoculation. The TATV-psoralen inoculated animal that did seroconvert suggests some virus particles were not fully inactivated and that measurement of virus infectivity is more sensitive in vivo compared to in vitro plaque assays which yielded no plaques (this finding is not unusual as IN and FP challenges of A-strain mice are lethal at doses <1 PFU which is below the threshold of detection for an in vitro plaque assay). All mice that were inoculated via the IN route with TATV-psoralen failed to seroconvert and experienced 100% mortality by Day 9 post inoculation with ECTV. These data suggest that the seroconversion experienced by animals is a function of TATV replication and not induced simply by the exposure of the immune system to TATV antigen. 

### 3.3. TATV Infection of Immunodeficient Mice

**Figure 1:**
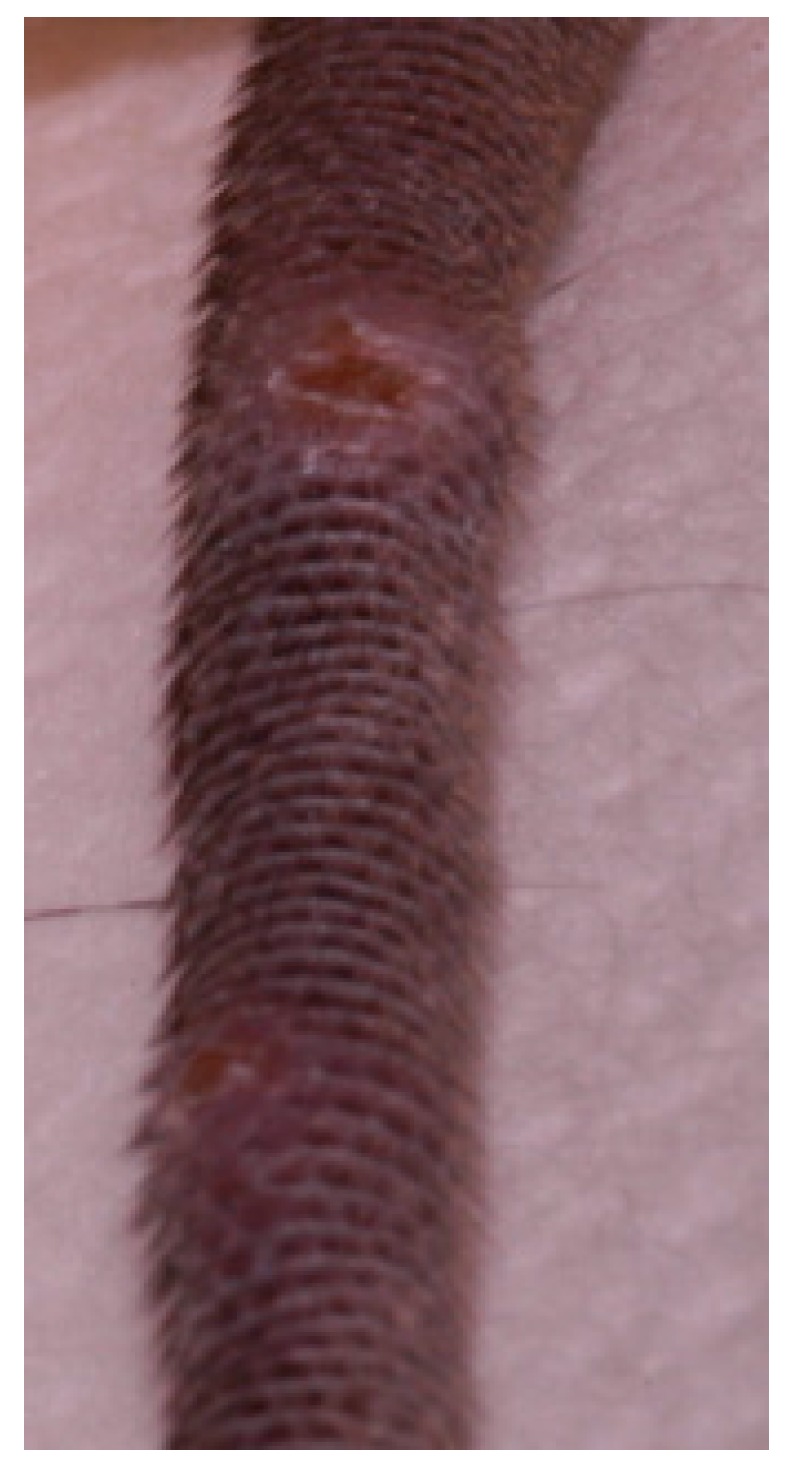
Typical tail lesion of a 129 *stat1*^−/−^ mouse inoculated via the FP with 1 × 10^6^ PFU of TATV. One mouse from total of *N* = 4.

Due to the failure to observe disease following infection of various immunocompetent species, we examined a number of immunocompromised murine strains. Murine strains lacking STAT1, a key protein involved in type 1 and type 2 interferon signaling networks, have been shown to be sensitive to a wide-number of viral and bacterial infections, including infections with MPXV [24]. 129 stat1^−/−^ mice inoculated with 1 × 10^6^ PFU of TATV via the FP route developed tail lesions from Day 16 p.i. By Days 25 and 39 p.i., 129 stat1^−/−^ mice had 5.5 ± 1.2 and 6.2 ± 1.8 lesions/tail, respectively. These lesions appeared along the tail, were discreet and reached a size of approximately 5 mm (Figure 1). Interestingly, C57BL/6 stat1^−/−^ mice failed to develop lesions; however, both strains developed severe FP swelling by Day 5 p.i. No other signs of morbidity were observed and all animals seroconverted by Day 60 p.i. Infections of 129 stat1^−/−^ and C57BL/6 stat1^−/−^ strains with 1 × 10^6^ PFU of TATV via the IN route resulted in no weight-loss, morbidity or mortality, although all inoculated mice seroconverted by 60 days p.i. (Table 1). Virus infectivity was not detected in livers, kidneys, spleens, lungs and blood from mice sacrificed on Days 6, 10 and 28 p.i. (data not shown). 

**Figure 2:**
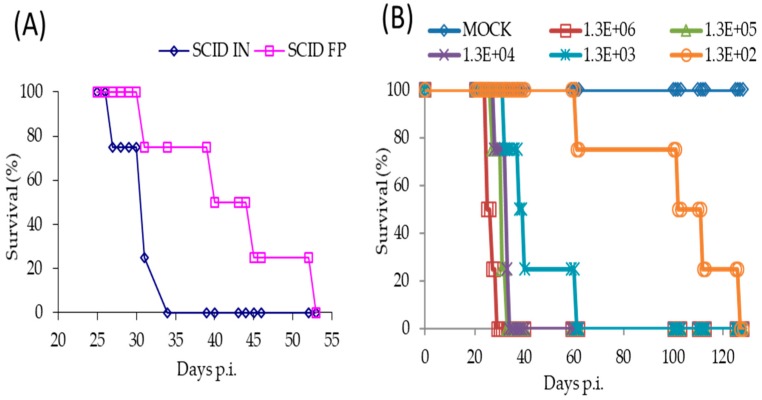

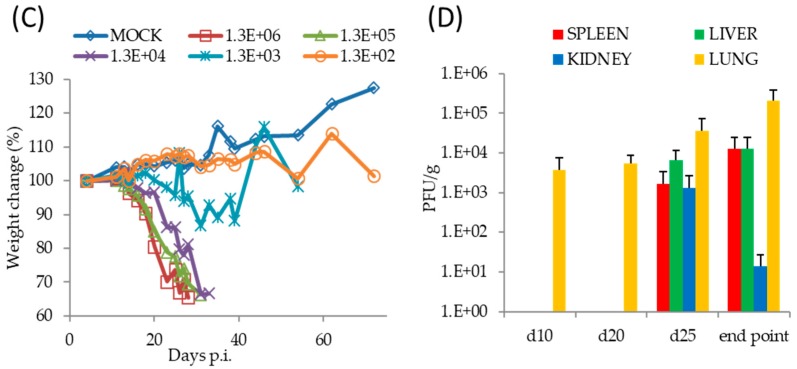
Pathogenesis of TATV in SCID mice infected by IN or FP routes. (**A**) SCID mice were inoculated with a 1 × 10^6^ PFU IN or FP dose of TATV and mortality rates were measured. SCID mice were inoculated via the IN route with TATV doses ranging from 1 × 10^6^ PFU to 1 × 10^2^ PFU and mortality rates (**B**) and weight-change as a percent of starting weight (**C**) were measured (standard error (SEM) bars are removed for clarity). (**D**) Tissue titers from the IN 1 × 10^6^ PFU inoculation were measured. *N* = 4 animals. Experiments were performed twice and typical results are shown.

Following a FP infection with 1 × 10^6^ PFU of TATV, we found SCID mice on a BALB/c background (SCID) and SCID mice on a hairless SKH1 background (SCID-SKH1) experienced 100% and 50% mortality by Day 52, respectively. Tail lesions were detected on SCID-SKH1 and SCID mice strains starting on Days 16 and 25 p.i., respectively. Because the SCID mice presented with the greatest mortality, we decided to further investigate pathogenesis following FP and IN infections of 1 × 10^6^ PFU of TATV (Figure 2). We found that IN inoculated mice experienced 100% mortality approximately 20 days earlier than those inoculated via the FP route (Table 1 and Figure 2A). To further explore the pathogenesis of IN inoculated mice, we inoculated groups of mice with TATV doses ranging from 1 × 10^6^ PFU down to 1 × 10^2^ PFU (Figure 2B). Mice inoculated with the lowest virus dose took until Day 127 p.i. to experience 100% mortality, whereas mice were dead by Day 29 p.i. at the highest dose (Figure 2B). Rate of weight-loss and total weight-loss (as a % of starting weight) was also a function of inoculation dose (Figure 2C). Following the 1 × 10^6^ PFU IN infection, virus was recovered from the lung by Day 10 p.i. but virus could not be detected in the spleen, liver and kidney until Day 25 p.i. (Figure 2D). We found no infectious virus in the blood at any of the time points (blood could not be removed from dead, non-sacrificed animals) and tail lesions were not apparent. Histological examination of mice inoculated with 1 × 10^6^ PFU and sacrificed at a moribund state revealed a reduction in splenic extramedullary hematopoiesis in TATV inoculated mice compared to mock animals. It also revealed that all TATV inoculated mice presented with fibrinopurulent rhinitis in the nasal passages and that all TATV inoculated animals had adrenal subcapsular cell hyperplasia. One TATV inoculated animal also presented with pancreatic lobular degeneration. All other observations were normal for SCID mice of the age used. Similar results were obtained following FP infection of SCID mice except the disease course was extended with significant weight-changes by Day 31 p.i. (117 ± 4.4% and 92.3 ± 2.9%, *p* = 0.009 for mock and inoculated groups, respectively). As expected, we also began to detect tail lesions on inoculated mice from Day 25 p.i.; these lesions increased in number until the day of death. Virus was detected in spleens and lungs from Day 20 p.i., in the liver from Day 30 p.i., in the kidney from Day 40 p.i. and in the blood from Day 48 p.i. (data not shown). The finding of infectious virus in the blood from the FP inoculated mice is in contrast to IN inoculated mice which presented with no infectious virus in the blood; however, consideration should be given to the fact that TATV was detected in the blood of FP inoculated mice that were in groups that were moribund or had already experienced mortality. In the IN inoculated mice, the final time point for blood sampling was from groups of mice that had not yet experienced any mortality and did not appear moribund. Therefore, it is possible that infectious virus in blood only appears a few days before death. Histologic findings following FP infection were similar to those following IN infection. The presence of virus in tissues was not associated with obvious pathology following both routes of infection as the major findings in sacrificed, moribund mice were a reduction of extramedullary hematopoiesis, a reduction in acute lung congestion, an increase in adrenal subcapsular cell hyperplasia, and mesenteric lymph node hypoplasia. All other observations were normal for SCID mice of the age used. These findings do not provide any explanation for cause of death.

### 3.4. Mortality in SCID Mice Is Not a Function of Virus Mutation

**Figure 3:**
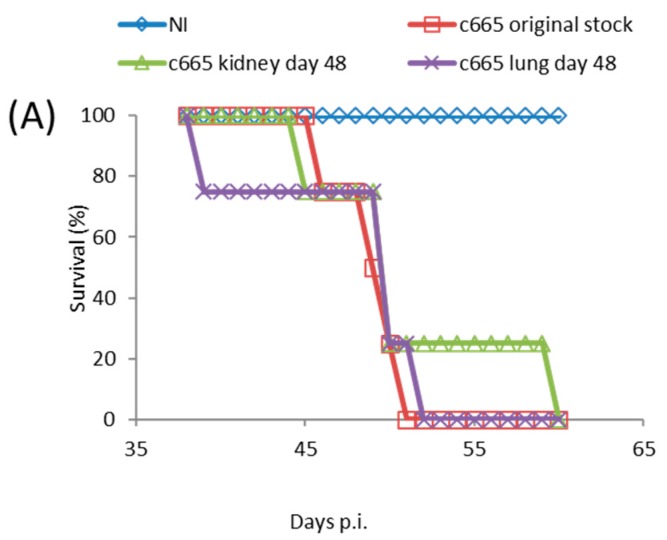

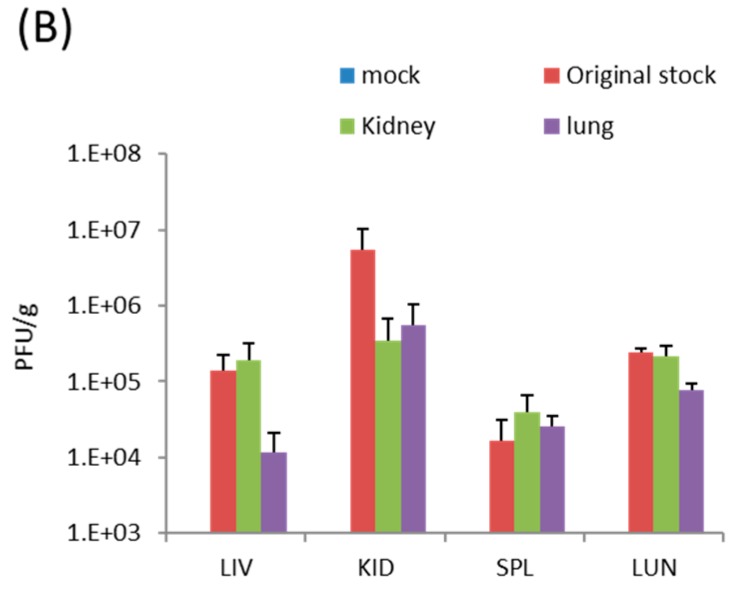
SCID mice were inoculated via the FP route with 1.7 × 10^4^ PFU of TATV recovered from the kidney and spleen of moribund mice (experiment #C665) following a 1 × 10^6^ PFU inoculation with TATV by FP the route. (**A**) Mortality; and (**B**) tissue titers from the livers, spleens and lungs of dead mice were measured. *N* = 4 mice.

TATV took significantly longer to kill SCID mice than is observed in ECTV and MPXV infections which kill all mice by Day 6 and Day 18, respectively [24]. The lack of detectable virus in tested tissues at 10 days p.i. from FP inoculated mice, and the progressive increase in tissue titers with time was consistent with the generation and/or selection of TATV mutants that were better adapted to replication in the mouse. If this hypothesis is true, the mutant virus would make-up a significant proportion of virus isolated at 48 days p.i., and infection of SCID mice with recovered virus would yield a shorter mean time to death than the original stock. Accordingly, groups of SCID mice were inoculated by the FP route with 1.7 × 10^4^ PFU of the original stock virus or kidney-lysate virus or lung-lysate virus from end-point specimens. We chose to take viral lysate from FP inoculated animals because they died at a later date and thus were provided more time for the selection of TATV mutations. Mice inoculated with the original stock virus experienced 100% mortality by Day 51 p.i., and mice inoculated with the kidney and lung-lysate virus reached 100% mortality on Day 52 and 60 p.i, respectively (Figure 3A). In addition, virus titers recovered from the spleens, livers, kidneys, and lungs of dead mice inoculated were similar (no significant different) between the inoculums (Figure 3B). Thus, the extended mean time of death of TATV-inoculated SCID mice is not consistent with the evolution of a virus strain with enhanced replication and/or virulence capacity in the mouse. Taken together, these studies in SCID mice suggest very inefficient replication and spread.

### 3.5. TATV Transmission

Because our failure to detect robust replication of TATV in tested immunocompetent animal models may be due to the failure to examine the relevant tissue, we utilized a transmission assay as another approach to detect biologically relevant virus infectivity. This assay evaluates virus shedding and determines if enough virus is produced for transmission. We tested both immunocompetent and immunocompromised mice for their ability to transmit TATV and we used ECTV as a transmission control as we have previously shown that ECTV is efficiently transmitted from an inoculated mouse to naïve cage mates over approximately a 10 day period [35]. As determined by seroconversion of contacts, we found that wild-type 129 and C57BL/6 mice transmitted ECTV when the index mouse was inoculated with 1 × 10^6^ PFU via the FP route; however, a similar experimental design with TATV-inoculated index mice did not detect seroconversion of contacts (Table 3). 129 stat1^−/−^ and C57BL/6 stat1^−/−^ mice inoculated with ECTV or TATV (1 × 10^6^ PFU) via the FP route failed to transmit the viruses. We next evaluated the transmission efficiency of SCID mice inoculated by the IN route—the route that facilitates increased TATV virulence. We found that SCID mice inoculated with 1 × 10^6^ PFU of TATV, but not ECTV, transmitted virus to C57BL/6 contact mice. The failure of SCID mice to transmit ECTV to C57BL/6 mice is likely due to the fact that the SCID mice die before virus titers reach a high enough level to infect contact mice. SCID mice inoculated by the IN route with ECTV or TATV successfully transmitted both viruses to C57BL/6 stat1^−/−^ mice, but only ECTV transmitted from the SCID mice to the A/Ncr contact mice (Table 3). Finally, we evaluated the ability of TATV to transmit from IN or FP inoculated gerbils and found that inoculated gerbils seroconverted by Day 70 p.i. but no seroconversion was detected in any of the contact animals (Table 3). These data reveal that TATV has reduced transmission capacity compared to ECTV. The ability of TATV to transmit to contact animals is only possible from inoculated, index SCID mice, and this finding is likely related to the fact that TATV replicates more efficiency in these animals—especially in lung tissue (Figure 2D).

### 3.6. Virus Replication and Cytokine Synthesis in the Primary Site of Infection (Footpad) and the Draining (Popliteal) Lymph Node

**Figure 4:**
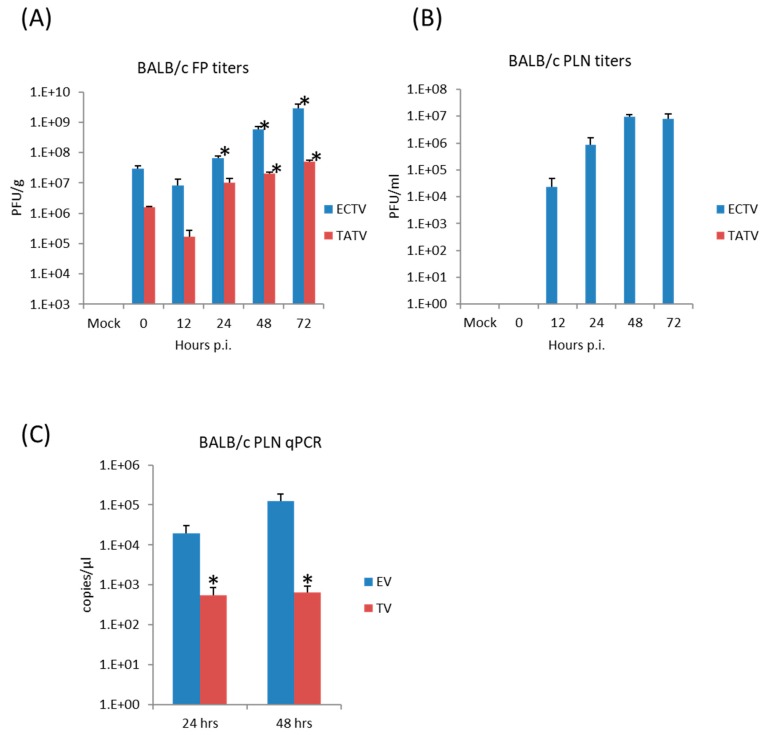
BALB/c mice were inoculated via the FP route with 1 × 10^6^ PFU of ECTV or TATV. The soft tissue of the FP was removed and titered for infectious virus up to 72 h p.i. (**A**); * indicates *p* < 0.05 increase in titers compared to the 0 h (input) titer for TATV or ECTV. Popliteal lymph node (PLN) titers (**B**); and levels of viral DNA (vDNA) (**C**) were determined; * indicates *p* < 0.05 lower levels of TATV compared to ECTV at 24 or 48 h p.i. Experiments were performed twice and typical results are shown. *N* = 4 animals.

**Figure 5:**
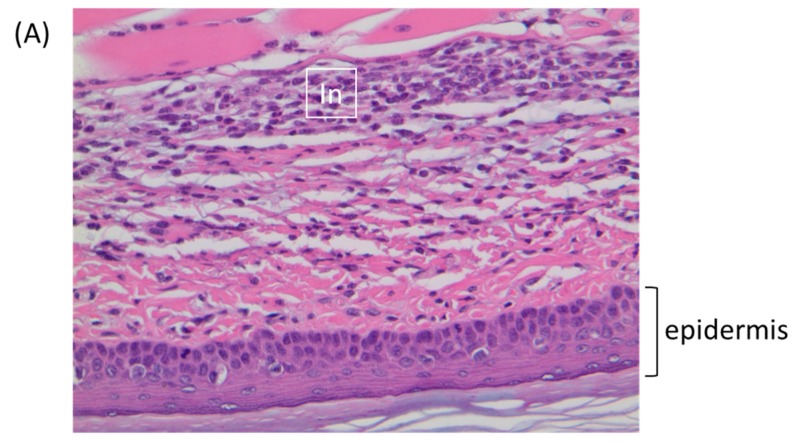

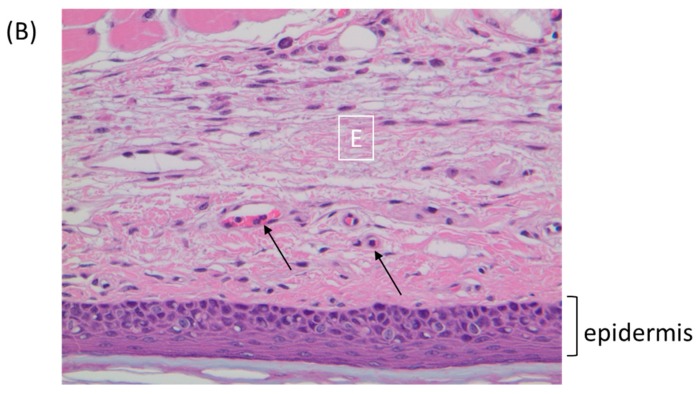
BALB/c mice were inoculated via the footpad with 1 × 10^6^ PFU of ECTV or TATV. At 72 h p.i., the feet were removed and sectioned along the mid-sagittal plane and stained with hematoxylin and eosin. (**A**) TATV infected footpads were marked by a mixed inflammatory cell infiltrate (In) in the superficial and deep dermis. (**B**) ECTV infected footpads presented with scattered mononuclear cells in the edematous dermis (E). Arrows indicate marginating neutrophils.

The FP route is the most thoroughly studied inoculation route in the ECTV/mousepox model [37,38,39]. We and others have shown that the inflammatory and early immune response in the lymph node draining the site of infection are critical for survival following ECTV infections via the FP route [38,40,41]. Accordingly, we examined virus replication and cytokine responses in the FP following inoculation of BALB/c mice with 1 × 10^6^ PFU of TATV or ECTV. To examine viral replication at the site of infection, the soft tissue of the FP was assayed for viral titers at 0 (input virus), 12, 24, 48, and 72 h p.i. We found that the input viral titer was higher for ECTV; however, we found that significant replication above input viral levels was detected in the ECTV FPs from 24 h p.i. and from 48 h p.i. in the TATV FPs (Figure 4A). For histological analysis, we removed the FP at 24 and 72 h p.i. and sectioned the mid-sagittal plane. At the 24 h time point, we could not observe any obvious differences between ECTV and TATV (data not shown) and at the 72 h time point we observed multifocal, subacute inflammation in the plantar metatarsal region of both ECTV and TATV samples; however, the severity was increased in TATV samples (Table 4). At the 72 h time point we could see mixed inflammatory cell infiltrate in the superficial and deep dermis of TATV inoculated animals whereas only scattered mononuclear cells in the edematous dermis along with the detection of marginating neutrophils were observed in the FP of ECTV inoculated mice. These findings suggest a more robust immune response to the presence of TATV at 72 h p.i. compared to ECTV at the same time (Figure 5) and may contribute to the increasing difference between ECTV and TATV titers. To further dissect virus dissemination, we looked at viral titers and viral DNA (vDNA) levels in the popliteal lymph node (PLN). Following a 1 × 10^6^ PFU inoculation, titers of TATV were not detected at 12, 24, 48 and 72 h p.i. in the PLNs, whereas levels of ECTV increased over the same 12–72 h time period (Figure 4B). As determined by qPCR, vDNA loads in the PLN at 24 and 48 h p.i. were detectable, but significantly lower in mice inoculated with TATV as compared to ECTV (Figure 4C). Similar findings were observed in the PLNs of SCID, A/Ncr and C57BL/6 mice inoculated with 1 × 10^6^ PFU of TATV or ECTV (data not shown) i.e., following a TATV inoculation vDNA was detected but the PLNs were negative for viral titers. In summary, these data suggest that TATV does not spread efficiently from the primary site of infection to the draining lymph node and that the innate immune response at the site of infection is far more robust in TATV inoculated animals compared to ECTV inoculated animals.

### 3.7. The Host Response at the Draining Lymph Node

The host response in the draining PLN was evaluated by measuring 32 different cytokines in mock, TATV and ECTV inoculated mice at 24 h p.i. Data are summarized in Table 5. As compared to controls, the levels of 19 cytokines were elevated, seven unchanged and one decreased in ECTV lysates, whereas three were elevated, 15 unchanged, and nine decreased in TATV lysates. The cytokine data in the PLN were consistent with a ramping up of a Th1-biased innate response to infection in the case of ECTV infections; however, TATV inoculations failed to broadly activate cytokines in the PLN.

## 4. Discussion

TATV was isolated in 1975, and yet, despite its close similarity to VARV, no research investigating its virology has been published in the last 42 years [28]. Two other studies have documented pox infections in gerbils. In 1971, Bradley et al. reported nodules on the tails of a gerbil, *T. robusta *(a close relative of *T. kempi*), in Uganda which presented with epidermal proliferation and a large number of intracellular poxvirus particles. Unfortunately, no further studies were published to characterize this virus [42]. In 1978, Marennikova et al. isolated a poxvirus from the kidney of a gerbil (*Rhombomys opimus*) from Turkmenia which was shown to cause a disease with high mortality in the gerbils as well as in Yellow Susliks (*Spermophilus* squirrels) via various inoculation routes; however, the virus isolate was shown to be very similar to CPXV in some lab assays and differs from TATV in its ability to infect mice at a low-dose inoculum [21].

We have conducted an investigation into the virology of TATV and can report that TATV cannot initiate robust disease in any tested immunocompetent murine strains, the Mongolian gerbil (*M. unguiculatus*), or the African dormouse (*G. kelleni*); however, as measured by seroconversion, TATV infects all these animals except for the dormouse. We confirm previous work by Lourie that the Mongolian gerbil presents with no obvious signs of disease—although Lourie et al. inoculated via the IC and IP routes [28], whereas we used the IN and FP routes. We also found that the gerbil fails to transmit TATV. A drawback to our gerbil studies was that they were conducted in the Mongolian gerbil, and TATV was isolated from an apparently healthy West African gerbil, *T. kempi*. These two gerbils are not closely related as they belong to different Tribes of the Subfamily *Gerbillinae* (Family *Muridae*): *T. kempi* is a member of the tribe *Taterillini*, and *M. unguiculatus* belongs to the tribe *Gerbillini* [43]. It would be interesting to investigate the virology of TATV in *T. kempi* but legal and logistical restrictions prevent the importation of these animals.

The finding that TATV can infect and produce lesions in immunodeficient *stat1*^−/−^ mice is important because it suggests that restriction is not merely a function of a lack of host-range ORFs. Furthermore, our data indicate that type 1 and 2 interferons are at some level required for controlling TATV infection. The transmission studies revealed that *stat1*^−/−^ mice could not transmit ECTV or TATV to other *stat1*^−/−^ mice. It was not overly surprising that ECTV, which is highly virulent in *stat1*^−/−^ mice, did not transmit to other *stat1*^−/−^ animals because the index mice died rapidly and most likely before virus could reach the extremities or mucus membranes—a finding we have observed in other ECTV studies (data not shown). Moreover, it is somewhat surprising that *stat1*^−/−^ mice did not transit TATV to other *stat1*^−/−^ mice given that the index animals would have experienced some morbidity and the development of tail lesions. The most likely explanation for this failure of transmission is that TATV in the index animals could not replicate to high enough titers to transmit to contact animals—a finding that is supported by the absence of tissue titers in *stat1*^−/−^ mice inoculated with TATV.

The only robust disease that we observed (other than tail lesions and swollen footpads) was in SCID mice on the SKH1 and BALB/c backgrounds. Of these we found the highest virulence in the BALB/c SCID mice, therefore confirming the importance of background genetics to the susceptibility of murine strains. This observation is not uncommon, for example A/Ncr and C57BL/6 mice present with different disease courses and mortality levels when infected with ECTV via the same route [38]. Furthermore, we found that the disease course was approximately 40% shorter in SCID mice infected via the IN route compared to the FP route. Again, this observation is not unusual as many strains of mice are susceptible to IN ECTV infection but are resistant to FP infections [39]. Other OPVs (CPXV, MPXV and VACV) have been evaluated in SCID mice and typically induce a highly fulminant disease with a mean time to death of less than 12 days [24,44,45,46,47]. Following FP infection of SCID mice, there was a progressive increase of TATV titers in spleen, liver, kidney, and lungs until death at ~50 days p.i., which was consistent with the generation and/or selection of TATV mutants of enhanced virulence; however, this explanation was ruled out experimentally by re-passage of kidney and lung lysates from TATV-infected SCID mice that also died at ~50 days following FP inoculation. Examination of histological evidence acquired from moribund SCID mice following an IN or FP inoculation failed to identify a plausible cause of death. In the case of smallpox, interstitial pneumonitis and tubulointerstitial nephritis are thought to be the main cause of death [48]; however, neither of these were identified in the TATV-infected SCID mice. Similarities also could not be drawn to other IN models of disease such as: VACV IN challenges of mice which present with necrotizing bronchopneumonia [49,50]; IN CPXV challenges where mice present with tracheitis/tracheobronchitis, bronchiolitis/bronchopneumonia, rhinitis/sinusitis, meningitis, cranial myositis, otitis media, and eustachitis [47,51]; and in aerosolized and IN MPXV challenges of cynomolgus macaques which die due to fibrinonecrotic bronchopneumonia [52,53].

We hypothesize that TATV fails to cause systemic disease in tested immunocompetent mouse strains due to a failure to optimally replicate, spread and counteract the innate immune response to infection. As compared to ECTV, which has previously been shown to replicate at the site of infection [54,55], TATV titers were reduced at the primary site of replication and the magnitude of the difference increased throughout the 72 h period of observation. This was consistent with an in vitro replication assays that found TATV had a diminished replication capacity in tested murine epithelial (key target cell of poxviruses) and fibroblast cell lines as compared to ECTV (data not shown). These in vitro findings will be addressed in a subsequent paper.

TATV infectivity was not detected in the PLN draining the primary site of replication in BALB/c or SCID mice up to 48 hours p.i. This was surprising as studies with VACV in mice [56], and ECTV in mice [54,57] have detected virus infectivity as early as 6–12 h p.i. The failure to detect TATV in the PLN was not due to the presumed lower TATV replication rate in skin as by 48 h p.i. the TATV skin titers were equal to that observed for ECTV at 12 h p.i., a time point when ECTV could be observed in the PLN. By 24 h p.i., dramatic upregulation of many cytokines was detected in the PLNs from ECTV inoculated mice; however, in TATV PLNs cytokines generally failed to respond or responded differently to those of ECTV infected PLNs. This lack of a change in the cytokine/chemokine pattern following TATV infection may be due to the failure of TATV to replicate robustly in the PLN or because TATV never arrives at the PLN. That said, TATV vDNA was detected at low levels in the PLN at 24 and 48 h p.i. The detection of vDNA but not viral antigen could explain the triggering of a different cytokine response compared to ECTV which replicates at the PLN with vDNA also detected. The cytokine responses at the PLN following ECTV infection are well studied. Parker et al. demonstrated that the PLNs of C57BL/6 and A-strain mice respond differently following a FP inoculation with ECTV with resistant C57BL/6 mice favoring a Th1 response compared to susceptible A-strain mice which favour a Th2 response [38]. Furthermore, Chaudhri et al. revealed a detailed breakdown of cytokine changes in the PLNs of susceptible and resistant mice following a FP inoculation with ECTV and confirm that resistance favors a Th1 cytokine response [58]. In this paper, we did not detect an obvious Th1 vs. Th2 response in BALB/c mice inoculated with TATV or ECTV via the FP route; however, our study was limited to a very early time point (up to 24 h p.i.).

In conclusion, TATV in the animals that we evaluated does not provide a suitable model for smallpox or human monkeypox. In vitro and bioinformatics studies (data not shown) suggest that, similar to VARV, TATV has a very narrow host-range. Whether the gerbil is the natural host of TATV remains to be elucidated. Although TATV does not replicate well in the immunocompetent animals that we evaluated, it did induce seroconversion. This finding suggests that TATV could be used as an OPV vaccine in animals or humans.

## Figures and Tables

**Table 1:** Inoculation of female mice, gerbils and dormice with 1 × 10^6^ plaque forming units (PFU) of Taterapox virus (TATV) ^1^.

**Immunocompetent**					
**Species/Strain**	**Route ^2^**	**Seroconvert ^3^**	**Mortality (%)**	**Day of Death**	**Weight Loss (%) ^5^**
Gerbil	IN/FP	+	0	N/A ^4^	-
Dormouse	IN/FP	−	0	N/A	-
A/Ncr	IN/FP	+	0	N/A	-
SKH-1	IN/FP	+	0	N/A	-
C57BL/6	IN/FP	+	0	N/A	-
CAST/EiJ	IN/FP	+	0	N/A	-
129	IN/FP	+	0	N/A	-
**Immunodeficient**					
**Species/Strain**	**Route**	**Seroconvert**	**Mortality (%)**	**Day of Death**	**Weight Loss (%) ^5^**
129 *stat1*^−/−^	IN/FP	+	0	N/A	-
C57BL/6 *stat1*^−/−^	IN/FP	+	0	N/A	-
SCID (BALB/c)	IN	N/A	100	31 ± 3	34.5
SCID (BALB/c)	FP	N/A	100	51 ± 5	28.3
SCID (SKH1)	FP	N/A	50	52 ± 0	15.8

^1^ Animals were kept on-study for 100 days. ^2^ Challenge route was either intranasal (IN) or footpad (FP). ^3^ Seroconvert by Day 60 p.i. ^4^ N/A, not applicable. ^5^ Maximum percentage weight loss compared to *T* = 0 weight.

**Table 2:** Seroconversion following IN or FP inoculation of BALB/c mice with TATV or TATV-psoralen ^1^.

Cage	Virus ^2^	TATV Inoculation Route (*T* = 0) ^2^	Mice Seroconversion (*T* = 28) ^3^	ECTV Inoculation Route (*T* = 35) ^4^	DOD Following ECTV Challenge ^5^
1	Mock	FP	−−−	FP	7,7,9
2	Mock	IN	−−−	IN	7,8,7
3	TV	FP	+++	FP	
4	TV-psoralen	FP	−−+ ^6^	FP	6,7,ND ^6^
5	TV	IN	+++	IN	
6	TV-psoralen	IN	−−−	IN	8,8,9

^1^ TATV was made replication-inactive by psoralen treatment (see methods). ^2^ Mice were inoculated at *T* = 0 days with TATV or TATV-psoralen via the intranasal (IN) or footpad (FP) routes (1 × 10^3^ PFU). ^3^ At *T* = 28 days mice were bled for ELISA to determine seroconversion; − indicates negative for antibodies; + indicates positive for antibodies. Scores are given for each mouse individually. ^4^ At *T* = 35 days mice were inoculated with a lethal dose of ectromelia (ECTV) via the corresponding IN or FP route (1 × 10^3^ PFU) and monitored for mortality. ^5^ Days of death (DOD) are indicated for each mouse post inoculation with ECTV. Individual days of death are recorded in the same order as seroconversion status is recorded (column 4). ND indicates no death. ^6^ One mouse seroconverted following FP TATV-psoralen inoculation and survived the subsequent ECTV inoculation on Day 35.

**Table 3:** Transmission of TATV and ECTV between index and contact mice following 1 × 10^6^ PFU challenges ^1^.

Index	Route	Contact	ECTV ^2^	TATV ^2^
129	FP	129	4/4	0/4
129 *stat1*^−/−^	FP	129 *stat1*^−/−^	0/4^3^	0/4
C57BL/6	FP	C57BL/6	4/4	0/4
C57BL/6 *stat1*^−/−^	FP	C57BL/6 *stat1*^−/−^	0/4 ^3^	0/4
SCID	IN	C57BL/6	0/4 ^3^	4/4 ^7^
SCID	IN	C57BL/6 *stat1*^−/−^	4/4 ^3,4^	4/4 ^7^
SCID	IN	A/Ncr	4/4 ^3,5^	0/4
Gerbil	FP	Gerbil	N/A ^6^	0/4
Gerbil	IN	Gerbil	N/A	0/4

^1^ Index mice were inoculated by IN or FP routes with 1 × 10^6^ PFU of ECTV or TATV and left isolated for 48 h. After 48 h, index mice were introduced to naïve contact mice (*N* = 4) for the remainder of the experiment. ^2^ Transmission determined by death and/or seroconversion by Day 70 p.i. ^3^ Index mice all dead by Day 8 p.i. ^4^ Contact mice all dead by Day 10 p.i. ^5^ Seventy-five percent of contact mice dead by Day 14 p.i. (survivors positive for seroconversion). ^6^ N/A, not applicable. ^7^ Transmission determined by seroconversion.

**Table 4:** Histopathology at 72 h p.i. of BALB/c feet inoculated via the FP route ^1^.

Virus	Diagnosis	Distribution ^2^	Severity ^3^	Number Presenting ^4^	Comments
TATV	Inflammation, subacute	MF	2	4	Plantar metatarsal region
	Inflammation, subacute	MF	3	2	Plantar metatarsal region
ECTV	Inflammation, subacute	MF	1	5	Plantar metatarsal region
	Inflammation, subacute	MF	1	1	Plantar metatarsal region and periosteal (distal tibia)

^1^ BALB/c mice (*N* = 3 per group for total of 6 feet) were inoculated via the FP with 1 × 10^6^ PFU of TATV or ECTV. At 72 h p.i., feet were removed to 10% neutral buffered formalin for 24 h and then moved to 70% ethanol prior to processing. ^2^ Distribution: F = Focal; MF = Multifocal; D = Diffuse. ^3^ Severity: 1 = Minimal; 2 = Mild; 3 = Moderate; 4 = Marked. ^4^ Number of samples presenting from total of six.

**Table 5:** Changes in cytokine levels at 24 h p.i. in the PLNs of BALB/c mice inoculated via the FP with 1 × 10^6^ PFU of ECTV or TATV ^1,2,3^.

Cytokine		ECTV			TATV		
Cytokine	Mock	↑ vs. Mock	≈ vs. Mock	↓ vs. Mock	↑ vs. Mock	≈ vs. Mock	↓ vs. Mock
Eotaxin	44 ± 4	125 ± 14				37 ± 4	
GM-CSF	9 ± 3	78 ± 11				4 ± 1	
M-CSF	20 ± 2	52 ± 4				30 ± 6	
TNF-α	5 ± 1	12 ± 1				6 ± 1	
IL-5	0.3 ± 0	10 ± 2				0.1 ± 0	
IL-13	43 ± 15	297 ± 39				15 ± 4	
MIP-1α	25 ± 4	44 ± 4				18 ± 3	
MIP-1β	48 ± 7	105 ± 11				46 ± 4	
IFN-γ	19 ± 5	81 ± 10				8 ± 2	
IP-10	91 ± 17	1232 ± 170				124 ± 31	
LIF	6 ± 2	42 ± 5				9 ± 2	
IL-12p70	3 ± 1	20 ± 4				1 ± 0	
MCP-1	58 ± 8	709 ± 60				116 ± 21	
MIG	949 ± 252	6896 ± 758				726 ± 116	
RANTES	79 ± 6	110 ± 7				76 ± 8	
G-CSF	251 ± 43		241 ± 40				89 ± 8
VEGF	16 ± 2		11 ± 2				6 ± 1
IL-10	11 ± 2		7 ± 1				3 ± 1
IL-12p40	230 ± 52		137 ± 18				64 ± 18
IL-1β	43 ± 6		44 ± 6				25 ± 3
IL-15	112 ± 22		93 ± 17				36 ± 9
IL-17	3 ± 1		4 ± 1				1 ± 0
IL-6	14 ± 3	189 ± 20			29 ± 3		
KC	23 ± 2	286 ± 25			56 ± 10		
IL-3	1 ± 0.1	7 ± 1			3 ± 0.4		
IL-9	532 ± 74			160 ± 44			145 ± 36
IL-1α	115 ± 16	228 ± 29					73.2 ± 7

^1^ BALB/c mice (*N* = 4) were inoculated via the footpad (FP) with 1 × 10^6^ PFU of ECTV or TATV. At 24 h p.i., the popliteal lymph node (PLN) was removed to phosphate buffered saline (PBS) with 0.05% Triton X-100 and ground up. The supernatant was removed and assayed for cytokines. ^2^ Unchanged cytokines (IL-2, IL-4, IL-7, MIP-2, and LIX) are not shown. ^3^ Cytokines in green are increased and cytokines in red are decreased, compared to mock (*p* < 0.05). GM-CSF: granulocyte macrophage colony- stimulating factor; TNF-α: tumor necrosis factor α; MIP: macrophage inflammatory protein; IL: interleukin; IFN-γ: interferon γ; LIF: leukemia-inhibitory factor; MCP-1: Monocyte chemoattractant protein-1; VEGF: vascular endothelial growth factor.
